# Transplantation Tolerance Induction: Cell Therapies and Their Mechanisms

**DOI:** 10.3389/fimmu.2016.00087

**Published:** 2016-03-07

**Authors:** Joseph R. Scalea, Yusuke Tomita, Christopher R. Lindholm, William Burlingham

**Affiliations:** ^1^Department of Surgery, Division of Transplantation, University of Wiconsin, Madison, WI, USA; ^2^School of Medicine, University of Wisconsin, Madison, WI, USA

**Keywords:** Treg, MDSC, regulatory mechanisms, regulatory myeloid cells, transplantation tolerance, HSCT, CD34^+^ cells

## Abstract

Cell-based therapies have been studied extensively in the context of transplantation tolerance induction. The most successful protocols have relied on transfusion of bone marrow prior to the transplantation of a renal allograft. However, it is not clear that stem cells found in bone marrow are required in order to render a transplant candidate immunologically tolerant. Accordingly, mesenchymal stem cells, regulatory myeloid cells, T regulatory cells, and other cell types are being tested as possible routes to tolerance induction, in the absence of donor-derived stem cells. Early data with each of these cell types have been encouraging. However, the induction regimen capable of achieving consistent tolerance, while avoiding unwanted sided effects, and which is scalable to the human patient, has yet to be identified. Here, we present the status of investigations of various tolerogenic cell types and the mechanistic rationale for their use in tolerance induction protocols.

## Introduction

Cell-based therapies lie at the root of transplantation tolerance induction protocols. Ray Owen at the University of Wisconsin made the early observation that a shared, naturally occurring neonatal blood supply was associated with the presence of chimeric red blood cell populations in adult cows (1). This, and other, observation prompted Peter Medawar to explore the possibility that donor chimerism would allow for acceptance of skin grafts from the same donor through which chimerism was established (2, 3). These findings, which led to the Noble Prize in 1960, were exploited by Dr. David Sachs (4) and Dr. Sam Strober (5) such that preclinical models (6) for tolerance to solid organ transplants could be developed (7–9). These preclinical models led to human clinical trials, which have since yielded encouraging results (10, 11).

Indeed, the mechanisms underlying tolerance development are still not clear. Since the completion of Medawar’s experiments, investigators have sought to identify the cell populations responsible for tolerance induction. Even today, however these cell types and their mechanisms remain elusive. Here, we will review some of the cell types, which have demonstrated tolerogenicity in both experimental and in preclinical models, focusing on the potential for tolerance induction in man.

## Donor Bone Marrow for Mixed Chimerism Establishment

Based on the notion that outcomes in human transplantation were unacceptable due to the requirement for long-term pharmacologic immunosuppression, and building on significant preclinical data, investigators at Massachusetts General Hospital attempted to achieve tolerance in humans. Their approach was to first establish lymphohematopoietic chimerism using the hematopoietic stem cells of the intended kidney donor, in order to establish a milieu where the donor and the recipient existed as a “mixed chimera” (10). In their seminal work published in the New England Journal of Medicine in 2008, investigators described the clinical course of five patients who received conditioning, bone marrow transplantation, and subsequent renal transplantation. Transplant recipients were conditioned using two preoperative doses of cyclophosphamide, as well as peri-transplantation anti-CD2, cyclosporine, and thymic irradiation. The five patients also underwent bone marrow transplantation and renal transplantation. In the group’s original description of the bone marrow procurement (11), investigators removed bone marrow from the donor’s iliac crest on the day of the transplant such that 2.7 × 10^8^ cells/kg were infused into an intended recipient (11).

As per their initial description, four of the five patients included in this study were tolerant, and off all immunosuppression at last recorded follow-up (between 2 and 5 years) (10). Interestingly, while chimerism was pan-detectable in the first week, four of five patients had no detectable chimerism as of day 14, and in the remaining one patient only 3.5% chimerism in the granulocyte lineage remained until day 21. In this respect, the attempt to achieve sustained mixed chimerism failed. Despite this, the authors observed excellent clinical results. Given the non-specific nature of the bone marrow transplantation, it is difficult to know what elements of the cell transplant (bone marrow in this case), conditioning regimen, and the organ itself in this early study were responsible for long-lasting tolerance. Irrespective of the mechanistic aspects of this initial study, these observations laid down the foundation for multiple pursuant studies, which have helped to address the tolerogenicity of cell-based transplants aimed at tolerance induction (10, 12).

Using donors and recipients who were HLA-matched siblings investigators at Stanford University employed a similar cell-based tolerance induction protocol for renal transplant recipients. Also published in the New England Journal of Medicine, Scandling et al. presented a series of 10 patients who underwent treatment with anti-thymocyte globulin, cyclosporine, and total lymphoid irradiation. Differing somewhat from the Massachusetts General Hospital (MGH) experience, an immunomagnetic bead column was used to enrich the bone marrow transplant for CD34^+^ hematopoietic stem cells. The bone marrow donor was first mobilized with a 5-day course of subcutaneous G-CSF 6 weeks prior to procurement. Their patient then received 8 × 10^6^ CD34^+^ hematopoietic stem cells in addition to 1 × 10^6^ CD3^+^ lymphocytes. The cell transplant was cryopreserved and administered on day 14, following completion of total lymphoid irradiation (13, 14). In more recent publications, the Stanford University group has shown that 8 of 15 patients completing the tolerance induction protocol were chimeric for 6 months or greater and successfully weaned from immunosuppression (14). Only four patients were not withdrawn from immunosuppression secondary to underlying disease or episodes or rejection (14). Thus, in a well-matched cohort, both sustained mixed chimerism and renal transplantation tolerance could be achieved using this approach.

A third group at Northwestern University has successfully implemented human tolerance induction protocols using a distinct, yet similar cell-based protocol. Again, T cell depletion was utilized, however with two doses of alemtuzumab (anti-CD52) (15, 16). Tacrolimus in addition to mycophenolate mofetil was initiated at the time of transplantation. The first of four bone marrow transfusions obtained via iliac crest aspiration were given on posttransplantation day 5, followed by repeat transfusions at months 3, 6, and 9 (16). Bone marrow donors were mobilized with Neuopogen prior to donation, and bone marrow infusions were enriched for CD34^+^ hematopoietic stem cells. Encouragingly, five of the institution’s first eight patients were stably tolerant of their renal allografts at 1-year posttransplantation (16). The Northwestern group has also employed the use of “facilitator cells” to augment the chimeric state and tolerogenic milieu, although the details of these CD8^+^ non-T cell types are largely unknown as they are considered proprietary (15, 17).

Taken together, it is clear that bone marrow infusions, likely through the action of CD34^+^ hematopoietic stem cells can lead to tolerance induction in humans. Importantly, and consistent with the initial observations of Starzl and Demetris (18), it may not be absolutely necessary for a high-level of chimerism to last indefinitely, in order for the transplanted graft to remain tolerated (8, 10, 12).

In fact, the loss of chimerism (>1% donor cells) may coincide with a totally chimerism-free state, wherein tolerance is sustained solely by anergy and immunoregulation induced by the kidney graft parenchyma, as suggested by Sachs et al. (7, 8) Alternatively, the loss of macro-chimerism may coincide with the onset of micro-chimerism (<0.1% donor cells), a setting in which the “two-way” model of transplant tolerance, as proposed by Starzl and Demetris, is sustained (18, 19). Although Starzl’s theory was based on mutual HvG/GvH reactions, and not on Regulatory T cells, a recent report indicates that Treg cells induced in the offspring during the transient chimerism stage of pregnancy are maintained by constant contact with rare maternal hematopoetic cells, indicating a key role for maternal microchimerism in tolerance (20).

In addition to the above descriptions of chimerism establishment, exciting new reports have promulgated an alternative hypothesis underlying the mechanisms of tolerance induction through bone marrow infusion. Authors have shown that CD34^+^ monocytes are capable of inducting apoptosis of donor reactive T cells, and that through Treg expansion, this leads to tolerance. Regardless of the underlying mechanisms, immune tolerance through bone marrow infusion has proven efficacy in humans. However, additional potentially less morbid cell-based therapies are in development as well (21).

## Mesenchymal Stem Cells

Adapted from bone-marrow transplantation efforts to reduce the rate of bone-marrow graft failure following haplo-identical transplantation, mesenchymal stem cells may be capable of tolerance induction (22, 23). Pluripotent mesenchymal stem cells are naturally occurring and exist within the bone marrow (24–28). Mesenchymal stem cells are precursors to bone, fat, and other connective tissues. Additionally, however, mesenchymal stem cells have been shown to support normal hematopoiesis and to demonstrate immunosuppressive qualities (22, 25, 27, 28). Mesenchymal stem cells can rapidly expand *ex vivo*, yet they do not lose potential to differentiate into multiple cell types (23, 24, 28). Partially explaining augmentation of haplo-identical bone-marrow transplantation, mesenchymal stem cells also assist with engraftment of hematopoietic stem cells (23).

It has been hypothesized that mesenchymal stem cells partly explain the tolerogenic nature of bone marrow transplantation for tolerance induction. Accordingly, small and large animal models of attempted tolerance induction using these cells have been studied (23). In a rodent model of heterotopic heart transplantation, investigators observed that rapamycin alone led to rejection of haplo-mismatched cardiac grafts by 3 weeks. In contrast, mesenchymal stem cell infusion as monotherapy inhibited acute rejection, and when infusion of mesenchymal stem cells was coadministered with rapamycin, recipients enjoyed long-term, and rejection-free graft survival (23). Recipients of mesenchymal stem cell infusion also displayed minimal antibody production. Investigators observed deposition of mesenchymal stem cells into the cardiac grafts, as well as increased number of FoxP3^+^ T regulatory cells (23). Mechanistically, authors offered that the intra-graft mesenchymal stem cells might (1) protect the donor heart from exposure of alloantigens, and (2) provide local immunomodulation for alloreactive T cell clones (23). While mesenchymal stem cells are certainly immunosuppressive, infusion of mesenchymal stem cells alone was insufficient to overcome the alloreactive host responses, suggesting that other factors intrinsic to the bone marrow (beyond mesenchymal stem cells) are potentially required for tolerance induction. Corroborating these findings, other authors have shown that mesenchymal stem cell infusions prolonged baboon skin graft survival (29) as well as survival of liver, kidney, and heart allografts in small animal models (23, 30–32).

The immunomodulatory effects of mesenchymal stem cells have been studied and their interplay with other immunological cell types has begun to be characterized (25–27). Indeed, authors have recently shown that the differential efficacy of mesenchymal stem cells is based on the cell source, suggesting that not all mesenchymal stem cells are created equally (33). While a complete understanding of the responsible mechanisms is incomplete, there is a clear upregulation of FoxP3^+^ Regulatory T cells resulting from mesenchymal stem infusion (34). In addition, the suppressive functions of mesenchymal stem cells are thought to be mediated by both cell-to-cell contact as well as through the action of soluble factors (35). Additionally, mesenchymal stem cells have been shown to down regulate MHC class II and costimulatory molecules, resulting expansion of regulatory dendritic cells and impaired alloreactive T cell homing, respectively (30, 35–37). Perhaps important to clinical applications, recent reports suggest that the timing of mesenchymal stem cell administration is important to graft survival. In addition, the immunosuppressive effects of mesenchymal stem cells have been shown to overcome the effects of graft versus host disease (GVHD) in man (38, 39). Indeed, in a rodent renal tolerance model, when mesenchymal stem cells were infused after kidney transplantation (versus prior), graft dysfunction and neutrophilic infiltration were observed within the graft. Unfortunately, however, at present it appears that the lifespan of mesenchymal stems cells is limited (28, 40). In contrast, significant graft survival prolongation was observed with the mesenchymal stem cell administration preceded organ transplantation (36). More recently, human studies of mesenchymal stem cell administration in living donor kidney transplantation demonstrated reduced doses of tacrolimus were required for those receiving cell therapy in addition to calcineurin inhibition (41), and improved graft function at 1 year. In 2015, investigators published of a human pilot study of renal transplantation, in which pre- and posttransplantation administration of autologous mesenchymal stem cells was found to be not only safe, but the infusion lead to upregulation of Tregulatory cells in recipients (42). Taken together, mesenchymal stem cells seem capable of significant immunosuppression; however, the immunosuppressive effects appear incomplete, suggesting that additional elements need to be addressed for tolerance induction via mesenchymal stem cell adminsitration (41, 43).

## *Ex vivo* Expanded Regulatory T Cells

### Regulatory T Cells

Regulatory T cells are perhaps the most widely discussed cell type with regard to tolerance induction and their biology has driven much of the recent research in transplantation tolerance (12, 13, 44–50). Regulatory T cells, of which there are many subsets, are naturally occurring, and are required for self-tolerance. Additionally, Regulatory T cells have been implicated in the immunosuppressive mechanisms described for each of the cell types presented in this manuscript (51–60). While some investigators have reasoned that Regulatory T cells may be a marker of tolerance rather than the unifying mechanism by which tolerance to organ transplants is mediated, few will argue with the idea that Regulatory T cells are critical to the success of tolerance protocols. Accordingly, recent data show that microchimerism may itself sustain antigen-specific Regulatory T cells in a mouse model (20). Indeed, the hypothesis that Regulatory T cells represent a marker of tolerance is gaining traction among the tolerance community (20).

From the standpoint of cell-based tolerance induction protocols, Regulatory T cells can be expanded *ex vivo* and administered exogenously, or transplanted as part of a tolerated graft (intra-graft Regulatory T cells; for caveats, see Section “Intragraft Regulatory T cells”). Endogenous Regulatory T cells have been studied extensively and are conventionally defined as thymic derived (tRegulatory T cells) or peripherally derived (pRegulatory T cells). tRegulatory T cells and pRegulatory T cells can be distinguished by different cell surface identifiers (CD39, CTLA-4, etc.) and by the soluble factors produced (IL-35, etc.). Notably, both tRegulatory T cells and pRegulatory T cells populations express intranuclear FoxP3, a transcription factor thought to be the most specific marker for Regulatory T cells (61–63). Additionally, helios, a member of the Ikaros family of transcription factors, has been shown to distinguish thymic from peripheral Regulatory T cells (64). While helios is expressed in 100% of thymocytes, naive rodent and human FoxP3 cells T cells generated peripherally via TCR stimulation failed to express helios (64). While the exact function of FoxP3 itself it not fully known, it is thought to downregulate the nuclear factor of activated T cells (NFAT) (62).

The mechanisms of Regulatory T cells have been extensively studied and recently reviewed (65). There are four primary actions, which are thought to mediate the inhibitory function of Regulatory T cells: (1) release of soluble, inhibitory factors, (2) cytolysis, (3) metabolic dysregulation, and (4) manipulation of the function of dendritic cells (65). The soluble factors IL-10 and TGF-beta have garnered significant interest in the Treg literature as the primary cytokines by which negative inhibition is mediated (66, 67). However, it is unclear if the cytokine profile for tRegulatory T cells and pRegulatory T cells is similar (65, 68). Building data from our laboratory and others have also suggested that IL-35 (Tomita et al., unpublished data) (69). It is also becoming clear that like natural killer cells and like cytotoxic T cells (CD8^+^), and regulatory T cells inhibit anti-donor responses via cytolysis through the activity of perforin and granzyme A (65, 70). While not widely discussed as a primary Treg function, regulatory T cells are also known to deplete IL-2 from the microenvironment, resulting in metabolic dysregulation of target T cells (71, 72). The interaction of Regulatory T cells and dendritic cells is bidirectional. Below in the review, we will discuss tolerogenic monocytes, which are upstream to Regulatory T cells, however Regulatory T cells themselves may also affect the maturation of suppressive monocytes through the action of CTLA-4 and other inhibitory signals (65, 73).

Given their known suppressive role *in vivo* following protocols of tolerance induction, much interest has focused on *ex vivo* expansion of Regulatory T cells such that subsequent administration might lead to tolerance induction. Regulatory T cells may be generated (induced Regulatory T cells or iRegulatory T cells) *ex vivo*, in the presence of IL-2 and TGF-beta (61, 67). Indeed preclinical and recent human trials have demonstrated that massive expansion of Regulatory T cells is possible, *ex vivo*. For such expansions, costimulation of purified Regulatory T cells (CD4^+^CD25^+^CD127^lo^) with CD28 in the presence of rapamycin has been associated with a 1000-fold increase in Regulatory T cells over approximately 3 weeks (74, 75). These protocols were extended to humans for the treatment of GVHD, with encouraging results. Notably, rapid expansion of Regulatory T cells *ex vivo* is associated with reduction is Regulatory T cells’ suppressive qualities, despite the production of FoxP3 (74, 75). Similar expansion rates (also using CD28 costimulation) and findings were observed in human studies of autoimmune hepatitis (76, 77) and other autoimmune diseases (51).

*Ex vivo* expansion of Regulatory T cells has been attempted in both preclinical and clinical settings (78, 79). In a mouse model, investigators were able to expand antigen-specific CD4^+^CD25^+^ Regulatory T cells using antigen-primed, immature dendritic cells (79). Authors then adoptively transferred these antigen-specific Regulatory T cells into skin-graft recipients (78). Investigators found that CFSE-labeled Regulatory T cells migrated into the transplanted grafts, that survival was prolonged (stable appearance and hear growth at >150 days), and that animals displayed evidence of transplantation tolerance (78). In a preclinical humanized mouse model of skin transplantation, investigators recently demonstrated that exogenous antigen-specific Treg administration significantly prolonged skin-graft survival. Importantly, the Treg expansion protocol utilizing CD69 and CD71 enrichment was thought to be scalable to the clinic (80). In a phase 1 2011 study, Regulatory T cells were expanded *ex vivo* from umbilical cord blood and administered to partially HLA-matched patients with hematologic malignancy. Not only did this prove to be safe but also it provided preliminary evidence that recipients of these Regulatory T cells had decreased risk of acute GVHD (59). Another 2011 study was able to show that Regulatory T cells coinfused with conventional T cells prevented GVHD without the use of posttransplant immunosuppressive therapy (60).

According to the National Institutes of Health, there are four open-active trials and one closed-active trial utilizing the infusion of *ex vivo* generated Regulatory T cells. A European group focused on cellular immunotherapy in organ transplantation has a phase 2 study in process in which autologous Regulatory T cells are removed from living donor renal transplant recipients, and after 5 days of expansion, they are reinfused into the recipient. In a second approved human trial, through the University of Minnesota, investigators are using autologous, donor alloantigen-specific Regulatory T cells produced from expanded Regulatory T cells obtained from pre-liver transplant patients. The Regulatory T cells are then infused back into the recipient at regular intervals with the goal of achieving tolerance. A group from the University of California San Francisco is using *ex vivo* generated and expanded Regulatory T cells to assess the effect on beta cell function and the autoimmune response in type 1 diabetes. Another phase 1 trial is investigating the safety, tolerability, and effect of three different doses of *ex vivo* expanded polyclonal Regulatory T cells in the cutaneous manifestation of patients affected with lupus erythematosus. Another phase 1 trial is using *ex vivo* Regulatory T cells for the prevention of acute GVHD in patients with hematological malignancies following hematopoietic stem cell transplantation. Another group from the University of California San Francisco is investigating the role of *ex vivo* expanded Regulatory T cells as a therapy for subclinical inflammation in kidney transplant patients.

## Intragraft Regulatory T Cells

It is widely accepted that immunomodulatory cell types home to areas of acute inflammation, and that these cell types establish a local, tolerogenic milieu (at least partly) through direct cell-to-cell interaction (44, 46, 48, 81–86). In a miniature swine animal model of MHC class-I disparate tolerance induction, authors have shown that a short course of calcineurin inhibition via cyclosporine leads to robust, long-lasting tolerance, which is not abrogated by infusion of pro-inflammatory cytokines, removal of the tolerated graft, or leukapheresis of peripheral T regulatory cells (44–46, 82, 87–89). Indeed, only when the tolerated kidney was removed for more than 3 months in this model, during which time the animal is kept alive by renal-transplantation with a recipient-matched kidney, did tolerance begin to wane (45, 90). These data are supported by mechanistic data in small animal models of heart transplantation (91). This abrogation of tolerance was hastened by sensitization with donor-derived peptide (45).

Given that Regulatory T cells are known to mediate both tolerance induction and tolerance maintenance in the model, investigators hypothesized that adoptive transfer of recipient-derived Regulatory T cells (both peripherally and from within the graft) could lead to stable tolerance in a naive recipient (44, 46). While adoptive transfer of leukapheresed Regulatory T cells alone did not lead to tolerance induction, transplantation of the tolerated kidney (with or without peripheral Treg infusion) did lead to stable tolerance in the naive recipient (44). These data suggested that the intra-graft regulatory components, widely thought to be CD4^+^CD25^+^FoxP3^+^ Regulatory T cells, were capable of overcoming the intrinsic alloreactive responses from the naive recipient (86, 88, 89, 92). In this way, adoptive transfer of intra-graft Regulatory T cells is thought to be capable of tolerance induction (46). While important mechanistically, this model itself has little direct applicability to the clinic. However, these data strongly support the notion that tolerance is mediated by immunoregulatory cells and that, were these cells clinically available, transplantation tolerance might be readily achieved. There are questions surrounding this cell population. For example, it is unclear what percentage of intagraft cells are antigen specific, in contrast to tRegulatory T cells and pRegulatory T cells. If, for example, intragraft Regulatory T cells are enriched with donor-specific Regulatory T cells, these mechanisms by which this occurs might be exploited and extrapolated to the clinic.

CD40L(CD154)/CD40 is one of the key costimulatory mechanisms required for T-cell activation. CD40L(CD154) monoclonal antibody has used as a blocker of this costimulation pathway. After the clinical failure of CD40L(CD154) blockade in humans and non-human primates (NHP), the interest in the CD40L(CD154)/CD40 axis has reemerged due to promising results with CD40 blockade. In mice, donor-specific transfusion (DST) plus CD40L(CD154) blockade is a standard and successful protocol to induce donor-specific transplant tolerance, involving apoptosis, acquisition of regulatory cells, and suppression of proliferation of effector cells (93, 94).

Abbas and colleagues (95) have shown that there can be many resident T cells in transplanted organs and tissues, including both pro-inflammatory memory T cells and memory Regulatory T cells. On day 30–40 after resolution of an inflammatory response in the skin, activated T cells, which had migrated from central lymphoid tissue, were maintained in the target tissue, thus developing “Treg memory” to that tissue. This period roughly corresponds to the kinetics of development of allo-specific, linked suppression responses observed in DST and CD40 blockade tolerization model (Tomita et al., submitted). Mechanistically, it is thought that anti-CD40L(CD154) leads to rapid changes in lymph node architecture and to the migration of Regulatory T cells and T effector cells through high-endothelial venules (96).

While capable of tolerance induction, the kinetics of peripheral allo-specific regulatory T memory cells into tissues (other than the lymphoid tissue) are unknown. In mice, approximately 5 weeks after DST and CD40 blockade, treatment was sufficient for allo-specific regulation to manifest itself in both the lymphoid tissue and the non-lymphoid organ (liver) (Tomita et al., submitted). The regulatory phenomenon was mediated by TGF-beta and IL-35, and the proportion of regulatory cytokine-producing CD4 T cells increased in lymphoid tissues and liver over time. However, TGF-beta producing and IL-35 producing cells had different migratory kinetics.

Whether Regulatory T cells (intra-graft or otherwise) induce tolerance directly or by virtue of facilitating other cell populations is unclear. Indeed, recently groups have reported that plasmacytoid dendritic cells are capable of facilitating hematopoietic cell engraftment. Below, we will address several addition cell populations, which may induce tolerance; however, it remains unclear if their function is by virtue of facilitation or by direct tolerogenic effects (17).

## Regulatory Myeloid Cells

Myeloid cells derive from hematopoietic stem cells. Rather than a rigidly defined group of progressively matured cell types, myeloid cells are better conceptualized as a network of cells, which can differentiate into various subsets (52). Regulatory myeloid cells (RMCs) include three broad classes of cells: regulatory macrophages (Mregs), dendritic regulatory cells (DCregs), and myeloid derived regulatory cells. *In vitro* models using human cells demonstrate each class of RMC can be generated from peripheral blood mononuclear cells (PBMCs) (58). However, the signals required for differentiation into each cell type (Mreg vs. DCreg vs. MDSC) are different. For example, *in vitro* differentiation of human PBMC into Mregs is facilitated by interferon gamma and macrophage colony stimulating factor (M-CSF). In contrast, expansion of DCregs from human PBMC is thought to require granulocyte/monocyte (GM)-CSF in addition to IL-4, IL-10, and TGF-beta plus other potentially tolerogenic factors. Lastly, MDSCs differentiation from PBMCs is supported by G-CSF and GM-CSF, and activation of MDSC requires IL-1, IL-6, and other pro-inflammatory factors (58).

Regulatory myeloid cells have elicited significant interest from the transplantation tolerance community, and clinical studies involving the use of DCregs as well as Mregs have been undertaken.

### Regulatory Macrophages

Regulatory macrophages are a uniquely characterized group of cells expressing a profile of distinct group of cellular markers. They possess a novel gene-expression profile that is different from monocytes, monocyte-derived DCs, resting macrophages, IFN-gamma stimulated macrophages, and M-1, M2a-, M2b-, and M2c-polarized macrophages (97). They are derived from peripherally isolated CD14^+^ monocytes that are cultured for 7 days while exposed to M-CSF, 10% human serum, and a 24-h pulse of IFN-gamma (98). The mechanisms by which these cells work have been investigated in both mice and humans. Mouse Mregs have been shown to inhibit T cell activity *in vitro* via inducible nitric oxide synthase (iNOS). In addition, Mregs delete cocultured allogeneic T cells via phagocytosis. In small animal models, T cells that avoided phagocytosis developed an impaired ability to secrete IL-2 and IFN-gamma (99). Human Mregs have been found to be potently suppressive of T cell proliferation via IFN-gamma induced indoleamine 2,3-dioxygenase (IDO) activity and contact-dependent deletion of activated T cells (100). Riquelme and colleagues were able to demonstrate that a one-time intravenous dose of donor-derived Mregs given 8 days before cardiac transplantation in mice was able to significantly prolong allograft survival in immunocompetent recipients. The graft survival was antigen-specific as graft survival. Indeed, recipient Mreg infusions (and third party controls) yielded no survival prolongation (99). This mechanism appeared to be iNOS independent.

Regulatory macrophages are an attractive option for cell-based tolerance induction in human recipients. A number of clinical trials have begun investigating this approach. The TAIC-I clinical trial was a single center, open-label single-arm study to assess the safety and tolerability of administering Mreg cell preparations to renal transplant recipients. A total of 12 patients receiving their first renal transplant from a decreased donor were enrolled and infused with 0.9–5.0 × 10^8^ cells via central venous access 5 days after transplantation. Mregs were isolated by culturing donor splenic mononuclear cells in M-CSF and stimulation with IFN-gamma. There were no acute or later observed adverse reactions, providing initial clinical evidence that this is a safe therapy (101). A subsequent trial, TAIC-II, assessed the safety and efficacy of administering Mreg cell preparations to recipients of living-donor renal transplants. A total of 5 living-related kidney transplant recipients were infused with 1.4–5.9 × 10^8^ cells, received induction therapy with anti-thymocyte globulin, in addition to steroid and tacrolimus (trough levels of 8–12 ng/ml). Mregs were obtained by culturing donor pPBMCs in M-CSF and stimulation with IFN-gamma followed by coculture with recipient PBMCs. No acute reactions occurred. Steroids were weaned by 8 weeks posttransplant, and tacrolimus was decreased to 5–8 ng/ml. Four patients were successfully transferred to this dose of tacrolimus therapy, with no rejection occurring in two patients. Tacrolimus levels were further weaned to <2 ng/ml, and one patient experienced rejection at 36 weeks. Following cessation of immunosuppression, two patients experienced rejection at 2 and 34 weeks postcessation (102). Another patient that did not qualify for the TAIC-II trial because of measurable levels of anti-donor HLA antibodies was described by Hutchinson and colleagues. The patient received a presensitized living-related renal transplant. The patient was infused with 4.8 × 10^9^ Mregs 17 days prior to transplant, which were isolated via the same protocol as the TAIC-II study. The patient was stable at 27 months posttransplant and interestingly was no longer positive for the anti-donor HLA antibodies. Serological screening determined that the patient remained hepatitis A virus positive (was positive before transplant) suggesting that this was a specific effect of Mreg treatment (103).

Since these two trials, Hutchinson and colleagues have refined their Mreg purification and treated two living-donor kidney transplant recipients. The first patient received a single HLA-B and DR mismatched-related kidney from her mother and 8 × 10^6^ donor-derived Mregs via central venous infusion 6 days prior to transplant. Azathioprine, steroids, and tacrolimus were started at the time of transplantation and at 3 years posttransplant, and the patient was stable with no signs of rejection demonstrated via biopsy while maintaining tacrolimus trough levels of 4–5 ng/ml. The second patient received a fully mismatched kidney from a living unrelated donor and 7.1 × 10^6^ Mregs 7 days prior to transplant. Azathioprine, steroids, and tacrolimus were started during transplantation. At 3 years posttransplant, the patient was stable with no signs of rejection via biopsy and was being maintained on tacrolimus with a trough level of 2.7 ng/ml (100). Taken together, preliminary evidence suggests that Mreg treatment preoperatively in renal transplant patients is safe, and further work needs to be done in humans to describe its effectiveness. The ONE Study is currently aiming to develop an array of cellular based therapies, one of which is Mregs, in order to achieve immunologic tolerance in transplant patients (104).

### Dendritic Regulatory Cells

Dendritic regulatory cells have been reviewed in detail recently (51, 97, 105). In one early human study of DCregs, authors observed that in response to injection of 2 × 10^6^ immature DCregs, antigen-specific Regulatory T cells were developed, and CD8^+^ T cell effector function was inhibited (58, 106, 107). Additionally, a more recent study of DCregs was undertaken in type I diabetes, for the purposes of self-tolerance (overcome autoimmunity). Authors administered 10 million cells intra-abdominally every 2 weeks for a total of four injections. DCreg injections were not associated with adverse reactions. Perhaps important, investigators did observe an increase in the percentage of suppressive B220^+^ B cells, which may help suppress autoimmunity in type 1 diabetes (108).

### MDSCs

MDSCs are a heterogeneous, immature population of monocytic- (mMDSCs) and granulocytic (gMDSCs)-derived cells that work to negatively regulate the immune system. MDSCs are naturally occurring, and are expanded during times of stress and inflammation (109). Much of what we know about MDSCs comes from cancer biology and the mechanisms by which MDSC-mediated immunosuppression occurs are being investigated. MDSC-mediated immunosuppression occurs through several known mechanisms. Primarily MDSCs have been found to express high levels of arginase-1 (produces urea and l-ornithine from l-arginine) and iNOS (generates NO), which have a well-established role in the suppression of T cell function (110, 111). By expressing arginase-1, MDSCs deplete local l-arginine levels of arginine, which is required by lymphocytes. In addition, MDSCs increase NO production. Arginase-1 dependent l-arginine depletion and NO production diminish the ability of T cells to proliferate and express MHC class II as well as inducing T cell apoptosis (112–116). MDSCs have also been shown to elicit immunosuppressive effects through the production of reactive oxygen species (ROS) and peroxynitrite (117–121). In the case of the latter, the peptide-MHC structure is altered, weakening the peptide’s immunogenicity (109). Likely important for potential tolerance induction, MDSCs have been found (in the presence of IFN-gamma and IL-10) to induce *de novo* development of FoxP3+ Regulatory T cells (116, 122). MDSCs are capable of inducing the proliferation of existing Regulatory T cells and that depletion of Trges impairs the ability of MDSCs to accumulate (116, 123, 124). The mechanisms by which MDSCs contribute to immune tolerance is multifactorial, involves other cell types and is likely to be subset dependent as well (109, 125).

With regard to MDSCs and solid organ transplantation, Vanhove and colleagues have shown in a kidney transplant rat model that immune tolerance was induced via anti-CD28 and that MDSCs accumulated within the allograft (126, 127). *In vitro*, the MDSCs were able to induce contact-dependent apoptosis of T cells, which induced the expression of iNOS in the MDSCs. The MDSCs were also found to have a minimal effect on Regulatory T cells that failed to induce iNOS in the MDSCs. These results highlight the cross-talk between these two cell types in immune tolerance. Lu et al. demonstrated that transplantation of hepatic stellate cells into diabetic mice induced MDSCs. In addition, these MDSCs were associated with increased levels of iNOS and Arg-1 as well as CD4^+^ and CD8^+^ T cell suppression. The same group also demonstrated that with cotransplantation of 2.5 × 10^6^ MDSCs and islet cells into diabetic mice, the survival of the islet cell allograft was significantly prolonged (128). *In vitro* and *in vivo* data both supported the necessity of the B7–H1 interaction for induction of Regulatory T cells involved in this process. Another study using repeated injections of LPS to induce MDSCs and evoke tolerance reported prolonged allograft survival through T cell suppression via a heme oxygenase-1 dependent pathway (129). This group was unable to reverse the T cell suppression by neutralizing iNOS or Arg-1, perhaps highlighting another immunomodulatory mechanism of MDSCs. Recently, Thomson and colleagues from the University of Pittsburgh showed that MDSCs can suppress T cell proliferation and cytokine secretion in non-NHP *in vivo* (130). This has raised the possibility of scaling these MDSC models to the NHP, and perhaps humans as well. In summary, much work is being done to uncover the mechanisms by which MDSCs contribute to establishing immune tolerance and the potential for use as a cellular based therapy is promising.

Regarding the potential for MDSCs in human transplantation, studies are lacking. Encouragingly, recent hematology data suggesting that MDCSs may control GVHD, and additional data demonstrating that MDSCs are upregulated after transplantation have highlighted MDSCs as a possible avenue to tolerance in humans (131). In a recent review, authors suggested that excitement for MDSCs in tolerance should be tempered until additional MDSC phenotyping can be performed. Indeed, it is not yet clear if the immunosuppressive effects of MDSCs are specific vs. non-specific, and it is not yet clear if MDSCs would need to be used synergistically with other therapies (127, 131).

## B Cells

While most studies have focused on the allo-reactive T cell in tolerance induction, the roles of allo-reactive B cells are largely unknown. However, a subset of B cells known as B regulatory cells (Bregs) has been identified as a potent factor in immune homeostasis and autoimmunity, and they have been found to be involved with maintaining immune tolerance associated with Regulatory T cells (132, 133). Recent work is uncovering a possible role in immunomodulation, which first gained attention when mice, deplete of B cells, were shown to develop a severe form of experimental autoimmune encephalomyelitis (EAE) (134). Further studies demonstrated similar findings in mouse models of autoimmune disorders such as collagen induced arthritis, ulcerative colitis, and allergy (135–138). In 2007, investigators at MGH (139) reported to achieve tolerance in a heart transplant mouse model. They first established B-cell dependent allo-reactive tolerance using anti-CD45RB antibody. The phenomenon required the interaction of costimulation molecules on B cells with T cells, which were CD40^+^ and CD80/86^+^. They also reported in islet allograft models that mice treated with anti-CD45RB antibody plus anti-T cells immunoglobulin domain and mucin domain-1 (anti-TIM-1) antibody were induced allo-reactive tolerance via an IL-10 dependent pathway (140). In addition, they recently showed that the Breg response was associated with Treg induction mediated by TGF-beta (141). A second group at University of Pittsburgh has indicated that TIM-1, which is an important marker for IL-10^+^ Bregs (induced by TIM-1 ligation), plays a critical role in regulation the immune response (142). A third group in Wisconsin has shown in an acute EAE mouse model deficient in B cells led to a delay in the emergence of FoxP3^+^ expression Regulatory T cells and the expression of IL-10 in the CNS. This was normalized by reconstitution with B cells, but was not normalized when reconstituted with B7 deficient B cells. The above work highlights a possible role for B cell dependent Treg expansion via B7 (143). Cell-to-cell contact has also been shown to contribute to B cell-dependent immunosuppression (144, 145). A recent study showed that coculture of purified Bregs was shown to suppress the proliferation of CD4^+^ T cells. Furthermore, Bregs coculture with Regulatory T cells led to the upregulation of FoxP3 and CTLA4 in Regulatory T cells (144). This evidence has led to the suggestion that Breg therapy may have an indirect role in immune tolerance therapy via *ex vivo* Treg expansion (133).

An immunoregulatory role for B cells has also been suggested in human diseases based on findings in patients with autoimmune diseases such as multiple sclerosis, lupus, rheumatoid arthritis, and even cancer (146–149). Numerous studies have begun to suggest that B cells also play an integral part in inducing immune tolerance in transplant patients (141, 150–154). Although there are no studies to date regarding B cell therapy in humans, this technique has been quite successful in animal models of autoimmune diseases. Particularly exciting is a model that has been developed in which polyclonal B cells are transduced with a retrovirus encoding specific antigens (155). Using this model, genetically modified B cells were able to inhibit autoimmune diseases such as uveitis, multiple sclerosis, type 1 diabetes, and rheumatoid arthritis in mouse models (19, 156–160). These genetically engineered B cells were also shown to be capable of inducing the proliferation of FoxP3^+^ CD4^+^ Regulatory T cells (161). Furthermore, another group was able to show that reconstitution with similarly engineered B cells *in vivo* protected against EAE in mice (162). Taken together, the success of B cell therapy for immunosuppression in animal models, and the established immunomodulatory role in humans suggests that the possibility of B cell-based cellular therapies for immune tolerance induction in humans is not out of the question.

## Other Cell Types

The above discussion is by no means complete. There are additional cell types which not included here which may be worthy of mention, such as apoptotic cells (163). Apoptotic cell-based therapies may improve graft survival and inflammatory diseases. Perhaps most excitingly, apoptotic cells may also be effective for the treatment of GVHD (163–165).

## Synthesis of the Data

Here, we have presented a number of different cell types, which contribute to tolerance induction. However, the presented data should be approached carefully. Indeed, mesenchymal stem cells or myeloid precursors (and/or MDSCs), which are present in the bone marrow may be involved in tolerance induction by cotransplantation of bone marrow and a solid organ. The same is true for facilitating cells. However, cell therapies based on regulatory T cells, B cells (Breg), dendritic cells, or macrophages emerge from their immunomodulatory properties rather than their sole presence in the bone marrow graft. Conversely, apoptotic cell-based therapies (i.e., administration of donor apoptotic cells) or facilitating cells may account for tolerance induction after cotransplantation of bone marrow and solid organ. As such, the notion of tolerance inducing versus tolerance facilitation may require further discussion.

## Author Contributions

JS – study design, analysis, literature review, wrote the paper, and edited the paper. YT – literature search, analysis, and wrote the paper. CL – literature search, analysis, and wrote the paper. WB – study design and wrote the paper.

## Conflict of Interest Statement

The authors declare that the research was conducted in the absence of any commercial or financial relationships that could be construed as a potential conflict of interest.

## References

[1] Owen RD. Immunogenetic consequences of vascular anastomoses between bovine twins. Science (1945) 102:400–1. 10.1126/science.102.2651.400 17755278

[2] Brent L. The discovery of immunologic tolerance. Hum Immunol (1997) 52:75–81. 10.1016/S0198-8859(96)00289-3 9077556

[3] Billingham RE Brent L Medawar PB. Actively acquired tolerance of foreign cells. Nature (1953) 172:603–6. 10.1038/172603a0 13099277

[4] Sharabi Y Sachs DH. Mixed chimerism and permanent specific transplantation tolerance induced by a nonlethal preparative regimen. J Exp Med (1989) 169:493–502. 10.1084/jem.169.2.493 2562984 PMC2189213

[5] Strober S Modry DL Hoppe RT Pennock JL Bieber CP Holm BI Induction of specific unresponsiveness to heart allografts in mongrel dogs treated with total lymphoid irradiation and antithymocyte globulin. J Immunol (1984) 132:1013–8. 6361129

[6] Sykes M. Mixed chimerism and transplant tolerance. Immunity (2001) 14:417–24. 10.1016/S1074-7613(01)00122-4 11336687

[7] Kawai T Cosimi AB Colvin RB Powelson J Eason J Kozlowski T Mixed allogeneic chimerism and renal allograft tolerance in cynomolgus monkeys. Transplantation (1995) 59:256–62. 10.1097/00007890-199501000-00018 7839449

[8] Kawai T Cosimi AB Wee SL Houser S Andrews D Sogawa H Effect of mixed hematopoietic chimerism on cardiac allograft survival in cynomolgus monkeys. Transplantation (2002) 73:1757–64. 10.1097/00007890-200206150-00011 12084998

[9] Kawai T Sogawa H Boskovic S Abrahamian G Smith RN Wee SL CD154 blockade for induction of mixed chimerism and prolonged renal allograft survival in nonhuman primates. Am J Transplant (2004) 4:1391–8. 10.1111/j.1600-6143.2004.00523.x 15307826

[10] Kawai T Cosimi AB Spitzer TR Tolkoff-Rubin N Suthanthiran M Saidman SL HLA-mismatched renal transplantation without maintenance immunosuppression. N Engl J Med (2008) 358:353–61. 10.1056/NEJMoa071074 18216355 PMC2819046

[11] Spitzer TR Delmonico F Tolkoff-Rubin N McAfee S Sackstein R Saidman S Combined histocompatibility leukocyte antigen-matched donor bone marrow and renal transplantation for multiple myeloma with end stage renal disease: the induction of allograft tolerance through mixed lymphohematopoietic chimerism. Transplantation (1999) 68:480–4. 10.1097/00007890-199908270-00006 10480403

[12] Kawai T Sachs DH Sykes M Cosimi AB Immune Tolerance N. HLA-mismatched renal transplantation without maintenance immunosuppression. N Engl J Med (2013) 368:1850–2. 10.1056/NEJMc1213779 23656665 PMC3760499

[13] Scandling JD Busque S Dejbakhsh-Jones S Benike C Millan MT Shizuru JA Tolerance and chimerism after renal and hematopoietic-cell transplantation. N Engl J Med (2008) 358:362–8. 10.1056/NEJMoa074191 18216356

[14] Scandling JD Busque S Dejbakhsh-Jones S Benike C Sarwal M Millan MT Tolerance and withdrawal of immunosuppressive drugs in patients given kidney and hematopoietic cell transplants. Am J Transplant (2012) 12:1133–45. 10.1111/j.1600-6143.2012.03992.x 22405058 PMC3338901

[15] Leventhal J Abecassis M Miller J Gallon L Ravindra K Tollerud DJ Chimerism and tolerance without GVHD or engraftment syndrome in HLA-mismatched combined kidney and hematopoietic stem cell transplantation. Sci Transl Med (2012) 4:124ra28. 10.1126/scitranslmed.3003509 22399264 PMC3610325

[16] Leventhal JR Mathew JM Salomon DR Kurian SM Friedewald JJ Gallon L Nonchimeric HLA-identical renal transplant tolerance: regulatory immunophenotypic/genomic biomarkers. Am J Transplant (2016) 16(1):221–34. 26227106 10.1111/ajt.13416 PMC4718825

[17] Fugier-Vivier IJ Rezzoug F Huang Y Graul-Layman AJ Schanie CL Xu H Plasmacytoid precursor dendritic cells facilitate allogeneic hematopoietic stem cell engraftment. J Exp Med (2005) 201:373–83. 10.1084/jem.20041399 15699072 PMC2213023

[18] Starzl TE Demetris AJ Transplantation tolerance, microchimerism, and the two-way paradigm. Theor Med Bioeth (1998) 19:441–55. 10.1023/A:1009924907775 10023193 PMC2993097

[19] Agarwal RK Kang Y Zambidis E Scott DW Chan CC Caspi RR. Retroviral gene therapy with an immunoglobulin-antigen fusion construct protects from experimental autoimmune uveitis. J Clin Invest (2000) 106:245–52. 10.1172/JCI9168 10903340 PMC517488

[20] Kinder JM Jiang TT Ertelt JM Xin L Strong BS Shaaban AF Cross-generational Reproductive fitness enforced by microchimeric maternal cells. Cell (2015) 162:505–15. 10.1016/j.cell.2015.07.006 26213383 PMC4522363

[21] D’Aveni M Rossignol J Coman T Sivakumaran S Henderson S Manzo T G-CSF mobilizes CD34+ regulatory monocytes that inhibit graft-versus-host disease. Sci Transl Med (2015) 7:281ra42. 10.1126/scitranslmed.3010435 25834108

[22] Ball LM Bernardo ME Roelofs H Lankester A Cometa A Egeler RM Cotransplantation of ex vivo expanded mesenchymal stem cells accelerates lymphocyte recovery and may reduce the risk of graft failure in haploidentical hematopoietic stem-cell transplantation. Blood (2007) 110:2764–7. 10.1182/blood-2007-04-087056 17638847

[23] Ge W Jiang J Baroja ML Arp J Zassoko R Liu W Infusion of mesenchymal stem cells and rapamycin synergize to attenuate alloimmune responses and promote cardiac allograft tolerance. Am J Transplant (2009) 9:1760–72. 10.1111/j.1600-6143.2009.02721.x 19563344

[24] Pittenger MF Mackay AM Beck SC Jaiswal RK Douglas R Mosca JD Multilineage potential of adult human mesenchymal stem cells. Science (1999) 284:143–7. 10.1126/science.284.5411.143 10102814

[25] Mattar P Bieback K. Comparing the immunomodulatory properties of bone marrow, adipose tissue, and birth-associated tissue mesenchymal stromal cells. Front Immunol (2015) 6:560. 10.3389/fimmu.2015.00560 26579133 PMC4630659

[26] Franquesa M Hoogduijn MJ Bestard O Grinyó JM. Immunomodulatory effect of mesenchymal stem cells on B cells. Front Immunol (2012) 3:212. 10.3389/fimmu.2012.00212 22833744 PMC3400888

[27] Engela AU Baan CC Dor FJ Weimar W Hoogduijn MJ. On the interactions between mesenchymal stem cells and regulatory T cells for immunomodulation in transplantation. Front Immunol (2012) 3:126. 10.3389/fimmu.2012.00126 22629256 PMC3355477

[28] Eggenhofer E Luk F Dahlke MH Hoogduijn MJ. The life and fate of mesenchymal stem cells. Front Immunol (2014) 5:148. 10.3389/fimmu.2014.00148 24904568 PMC4032901

[29] Bartholomew A Sturgeon C Siatskas M Ferrer K McIntosh K Patil S Mesenchymal stem cells suppress lymphocyte proliferation in vitro and prolong skin graft survival in vivo. Exp Hematol (2002) 30:42–8. 10.1016/S0301-472X(01)00769-X 11823036

[30] Casiraghi F Azzollini N Cassis P Imberti B Morigi M Cugini D Pretransplant infusion of mesenchymal stem cells prolongs the survival of a semiallogeneic heart transplant through the generation of regulatory T cells. J Immunol (2008) 181:3933–46. 10.4049/jimmunol.181.6.3933 18768848

[31] Wang Y Zhang A Ye Z Xie H Zheng S. Bone marrow-derived mesenchymal stem cells inhibit acute rejection of rat liver allografts in association with regulatory T-cell expansion. Transplant Proc (2009) 41:4352–6. 10.1016/j.transproceed.2009.08.072 20005397

[32] Ge W Jiang J Arp J Liu W Garcia B Wang H. Regulatory T-cell generation and kidney allograft tolerance induced by mesenchymal stem cells associated with indoleamine 2,3-dioxygenase expression. Transplantation (2010) 90:1312–20. 10.1097/TP.0b013e3181fed001 21042238

[33] Collins E Gu F Qi M Molano I Ruiz P Sun L Differential efficacy of human mesenchymal stem cells based on source of origin. J Immunol (2014) 193:4381–90. 10.4049/jimmunol.1401636 25274529 PMC4201962

[34] Qi H Chen G Huang Y Si Z Li J. Foxp3-modified bone marrow mesenchymal stem cells promotes liver allograft tolerance through the generation of regulatory T cells in rats. J Transl Med (2015) 13:274. 10.1186/s12967-015-0638-2 26293578 PMC4545923

[35] Uccelli A Moretta L Pistoia V. Mesenchymal stem cells in health and disease. Nat Rev Immunol (2008) 8:726–36. 10.1038/nri2395 19172693

[36] Casiraghi F Azzollini N Todeschini M Cavinato RA Cassis P Solini S Localization of mesenchymal stromal cells dictates their immune or proinflammatory effects in kidney transplantation. Am J Transplant (2012) 12:2373–83. 10.1111/j.1600-6143.2012.04115.x 22642544

[37] English K Barry FP Mahon BP. Murine mesenchymal stem cells suppress dendritic cell migration, maturation and antigen presentation. Immunol Lett (2008) 115:50–8. 10.1016/j.imlet.2007.10.002 18022251

[38] Le Blanc K Rasmusson I Sundberg B Götherström C Hassan M Uzunel M Treatment of severe acute graft-versus-host disease with third party haploidentical mesenchymal stem cells. Lancet (2004) 363:1439–41. 10.1016/S0140-6736(04)16104-7 15121408

[39] Le Blanc K Frassoni F Ball L Locatelli F Roelofs H Lewis I Mesenchymal stem cells for treatment of steroid-resistant, severe, acute graft-versus-host disease: a phase II study. Lancet (2008) 371:1579–86. 10.1016/S0140-6736(08)60690-X 18468541

[40] Eggenhofer E Benseler V Kroemer A Popp FC Geissler EK Schlitt HJ Mesenchymal stem cells are short-lived and do not migrate beyond the lungs after intravenous infusion. Front Immunol (2012) 3:297. 10.3389/fimmu.2012.00297 23056000 PMC3458305

[41] Peng Y Ke M Xu L Liu L Chen X Xia W Donor-derived mesenchymal stem cells combined with low-dose tacrolimus prevent acute rejection after renal transplantation: a clinical pilot study. Transplantation (2013) 95:161–8. 10.1097/TP.0b013e3182754c53 23263506

[42] Mudrabettu C Kumar V Rakha A Yadav AK Ramachandran R Kanwar DB Safety and efficacy of autologous mesenchymal stromal cells transplantation in patients undergoing living donor kidney transplantation: a pilot study. Nephrology (Carlton) (2015) 20:25–33. 10.1111/nep.12338 25230334

[43] Tan J Wu W Xu X Liao L Zheng F Messinger S Induction therapy with autologous mesenchymal stem cells in living-related kidney transplants: a randomized controlled trial. JAMA (2012) 307:1169–77. 10.1001/jama.2012.316 22436957

[44] Scalea JR Okumi M Villani V Shimizu A Nishimura H Gillon BC Abrogation of renal allograft tolerance in MGH miniature swine: the role of intra-graft and peripheral factors in long-term tolerance. Am J Transplant (2014) 14(9):2001–10. 10.1111/ajt.12816 25100613 PMC4194165

[45] Weiner J Scalea J Ishikawa Y Okumi M Griesemer A Hirakata A Tolerogenicity of donor major histocompatibility complex-matched skin grafts in previously tolerant Massachusetts general hospital miniature swine. Transplantation (2012) 94:1192–9. 10.1097/TP.0b013e31827254f5 23269447 PMC3531825

[46] Okumi M Scalea JR Gillon BC Tasaki M Villani V Cormack T The induction of tolerance of renal allografts by adoptive transfer in miniature swine. Am J Transplant (2013) 13:1193–202. 10.1111/ajt.12194 23464595 PMC3671754

[47] Sachs DH Kawai T Sykes M. Induction of tolerance through mixed chimerism. Cold Spring Harb Perspect Med (2014) 4:a015529. 10.1101/cshperspect.a015529 24384815 PMC3869282

[48] Hongo D Tang X Baker J Engleman EG Strober S. Requirement for interactions of natural killer T cells and myeloid-derived suppressor cells for transplantation tolerance. Am J Transplant (2014) 14:2467–77. 10.1111/ajt.12914 25311657 PMC4205183

[49] Sullivan JA Adams AB Burlingham WJ. The emerging role of TH17 cells in organ transplantation. Transplantation (2014) 97:483–9. 10.1097/TP.0000000000000000 24212502

[50] Dutta P Burlingham WJ. Microchimerism: tolerance vs. sensitization. Curr Opin Organ Transplant (2011) 16:359–65. 10.1097/MOT.0b013e3283484b57 21666480 PMC3337767

[51] Conde P Rodriguez M van der Touw W Jimenez A Burns M Miller J DC-SIGN(+) macrophages control the induction of transplantation tolerance. Immunity (2015) 42:1143–58. 10.1016/j.immuni.2015.05.009 26070485 PMC4690204

[52] Gabrilovich DI Ostrand-Rosenberg S Bronte V. Coordinated regulation of myeloid cells by tumours. Nat Rev Immunol (2012) 12:253–68. 10.1038/nri3175 22437938 PMC3587148

[53] Lucas CL Workman CJ Beyaz S LoCascio S Zhao G Vignali DA LAG-3, TGF-beta, and cell-intrinsic PD-1 inhibitory pathways contribute to CD8 but not CD4 T-cell tolerance induced by allogeneic BMT with anti-CD40L. Blood (2011) 117:5532–40. 10.1182/blood-2010-11-318675 21422469 PMC3109721

[54] Hutchinson JA Riquelme P Geissler EK. Human regulatory macrophages as a cell-based medicinal product. Curr Opin Organ Transplant (2012) 17:48–54. 10.1097/MOT.0b013e32834ee64a 22186091

[55] Hutchinson JA. Somatic cell-based therapy. Transplantation (2015) 99:1103–5. 10.1097/TP.0000000000000788 26035271

[56] Broichhausen C Riquelme P Geissler EK Hutchinson JA. Regulatory macrophages as therapeutic targets and therapeutic agents in solid organ transplantation. Curr Opin Organ Transplant (2012) 17:332–42. 10.1097/MOT.0b013e328355a979 22790067

[57] Ochando JC Turnquist HR. Innate immune cell collaborations instigate transplant tolerance. Am J Transplant (2014) 14:2441–3. 10.1111/ajt.12912 25311574

[58] Rosborough BR Raich-Regue D Turnquist HR Thomson AW. Regulatory myeloid cells in transplantation. Transplantation (2014) 97:367–79. 10.1097/TP.0b013e3182a860de 24092382 PMC3944083

[59] Brunstein CG Miller JS Cao Q McKenna DH Hippen KL Curtsinger J Infusion of ex vivo expanded T regulatory cells in adults transplanted with umbilical cord blood: safety profile and detection kinetics. Blood (2011) 117:1061–70. 10.1182/blood-2010-07-293795 20952687 PMC3035067

[60] Di Ianni M Falzetti F Carotti A Terenzi A Castellino F Bonifacio E Tregs prevent GVHD and promote immune reconstitution in HLA-haploidentical transplantation. Blood (2011) 117:3921–8. 10.1182/blood-2010-10-311894 21292771

[61] Safinia N Scotta C Vaikunthanathan T Lechler RI Lombardi G. Regulatory T cells: serious contenders in the promise for immunological tolerance in transplantation. Front Immunol (2015) 6:438. 10.3389/fimmu.2015.00438 26379673 PMC4553385

[62] Dummer CD Carpio VN Goncalves LF Manfro RC Veronese FV. FOXP3+ regulatory T cells: from suppression of rejection to induction of renal allograft tolerance. Transpl Immunol (2012) 26:1–10. 10.1016/j.trim.2011.08.009 21939765

[63] Veronese F Rotman S Smith RN Pelle TD Farrell ML Kawai T Pathological and clinical correlates of FOXP3+ cells in renal allografts during acute rejection. Am J Transplant (2007) 7:914–22. 10.1111/j.1600-6143.2006.01704.x 17286616

[64] Thornton AM Korty PE Tran DQ Wohlfert EA Murray PE Belkaid Y Expression of Helios, an Ikaros transcription factor family member, differentiates thymic-derived from peripherally induced Foxp3+ T regulatory cells. J Immunol (2010) 184:3433–41. 10.4049/jimmunol.0904028 20181882 PMC3725574

[65] Vignali DA Collison LW Workman CJ. How regulatory T cells work. Nat Rev Immunol (2008) 8:523–32. 10.1038/nri2343 18566595 PMC2665249

[66] Hara M Kingsley CI Niimi M Read S Turvey SE Bushell AR IL-10 is required for regulatory T cells to mediate tolerance to alloantigens in vivo. J Immunol (2001) 166:3789–96. 10.4049/jimmunol.166.6.3789 11238621

[67] Fu S Zhang N Yopp AC Chen D Mao M Chen D TGF-beta induces Foxp3 + T-regulatory cells from CD4 + CD25 – precursors. Am J Transplant (2004) 4:1614–27. 10.1111/j.1600-6143.2004.00566.x 15367216

[68] Shevach EM. From vanilla to 28 flavors: multiple varieties of T regulatory cells. Immunity (2006) 25:195–201. 10.1016/j.immuni.2006.08.003 16920638

[69] Collison LW Workman CJ Kuo TT Boyd K Wang Y Vignali KM The inhibitory cytokine IL-35 contributes to regulatory T-cell function. Nature (2007) 450:566–9. 10.1038/nature06306 18033300

[70] Velaga S Ukena SN Dringenberg U Alter C Pardo J Kershaw O Granzyme A is required for regulatory T-cell mediated prevention of gastrointestinal graft-versus-host disease. PLoS One (2015) 10:e0124927. 10.1371/journal.pone.0124927 25928296 PMC4415808

[71] Geis AL Fan H Wu X Wu S Huso DL Wolfe JL Regulatory T-cell response to enterotoxigenic bacteroides fragilis colonization triggers IL17-dependent colon carcinogenesis. Cancer Discov (2015) 5:1098–109. 10.1158/2159-8290.CD-15-0447 26201900 PMC4592451

[72] Garcia-Martinez K Leon K. Modeling the role of IL2 in the interplay between CD4+ helper and regulatory T cells: studying the impact of IL2 modulation therapies. Int Immunol (2012) 24:427–46. 10.1093/intimm/dxr120 22371423

[73] Kleijwegt FS Laban S Duinkerken G Joosten AM Koeleman BP Nikolic T Transfer of regulatory properties from tolerogenic to proinflammatory dendritic cells via induced autoreactive regulatory T cells. J Immunol (2011) 187:6357–64. 10.4049/jimmunol.1101638 22084438

[74] Golovina TN Mikheeva T Suhoski MM Aqui NA Tai VC Shan X CD28 costimulation is essential for human T regulatory expansion and function. J Immunol (2008) 181:2855–68. 10.4049/jimmunol.181.4.2855 18684977 PMC2556987

[75] Hippen KL Merkel SC Schirm DK Sieben CM Sumstad D Kadidlo DM Massive ex vivo expansion of human natural regulatory T cells (T(regs)) with minimal loss of in vivo functional activity. Sci Transl Med (2011) 3:83ra41. 10.1126/scitranslmed.3001809 21593401 PMC3551476

[76] Longhi MS Meda F Wang P Samyn M Mieli-Vergani G Vergani D Expansion and de novo generation of potentially therapeutic regulatory T cells in patients with autoimmune hepatitis. Hepatology (2008) 47:581–91. 10.1002/hep.22071 18220288

[77] Lapierre P Lamarre A. Regulatory T cells in autoimmune and viral chronic hepatitis. J Immunol Res (2015) 2015:479703. 10.1155/2015/479703 26106627 PMC4464004

[78] Golshayan D Jiang S Tsang J Garin MI Mottet C Lechler RI. In vitro-expanded donor alloantigen-specific CD4+CD25+ regulatory T cells promote experimental transplantation tolerance. Blood (2007) 109:827–35. 10.1182/blood-2006-05-025460 17003369

[79] Jiang S Camara N Lombardi G Lechler RI. Induction of allopeptide-specific human CD4+CD25+ regulatory T cells ex vivo. Blood (2003) 102:2180–6. 10.1182/blood-2003-04-1164 12775574

[80] Sagoo P Ali N Garg G Nestle FO Lechler RI Lombardi G. Human regulatory T cells with alloantigen specificity are more potent inhibitors of alloimmune skin graft damage than polyclonal regulatory T cells. Sci Transl Med (2011) 3:83ra42. 10.1126/scitranslmed.3002076 21593402 PMC3776382

[81] Bettini M Vignali DA. Regulatory T cells and inhibitory cytokines in autoimmunity. Curr Opin Immunol (2009) 21:612–8. 10.1016/j.coi.2009.09.011 19854631 PMC2787714

[82] Rosengard BR Fishbein JM Gianello P Ojikutu CA Guzzetta PC Smith CV Retransplantation in miniature swine. Lack of a requirement for graft adaptation for maintenance of specific renal allograft tolerance. Transplantation (1994) 57:794–9. 10.1097/00007890-199403270-00003 8154022

[83] Utsugi R Barth RN Lee RS Kitamura H LaMattina JC Ambroz J Induction of transplantation tolerance with a short course of tacrolimus (FK506): I. Rapid and stable tolerance to two-haplotype fully mhc-mismatched kidney allografts in miniature swine. Transplantation (2001) 71:1368–79. 10.1097/00007890-200105270-00003 11391221

[84] Scalea JR Bromberg J Bartlett ST Scalea TM. Mechanistic similarities between trauma, atherosclerosis, and other inflammatory processes. J Crit Care (2015) 30(6):1344–8. 10.1016/j.jcrc.2015.07.024 26304513

[85] Pillai AB George TI Dutt S Strober S. Host natural killer T cells induce an interleukin-4-dependent expansion of donor CD4+CD25+Foxp3+ T regulatory cells that protects against graft-versus-host disease. Blood (2009) 113:4458–67. 10.1182/blood-2008-06-165506 19221040 PMC2676099

[86] Griesemer AD Lamattina JC Okumi M Etter JD Shimizu A Sachs DH Linked suppression across an MHC-mismatched barrier in a miniature swine kidney transplantation model. J Immunol (2008) 181:4027–36. 10.4049/jimmunol.181.6.4027 18768858 PMC2694842

[87] Rosengard BR Ojikutu CA Guzzetta PC Smith CV Sundt TM III Nakajima K Induction of specific tolerance to class I-disparate renal allografts in miniature swine with cyclosporine. Transplantation (1992) 54:490–7. 10.1097/00007890-199209000-00020 1412729

[88] Gianello PR Lorf T Yamada K Fishbein JM Nickeleit V Vitiello DM Induction of tolerance to renal allografts across single-haplotype MHC disparities in miniature swine. Transplantation (1995) 59:884–90. 10.1097/00007890-199503150-00023 7701585

[89] Gianello PR Fishbein JM Rosengard BR Lorf T Vitiello DM Arn JS Tolerance to class I-disparate renal allografts in miniature swine. Maintenance of tolerance despite induction of specific antidonor CTL responses. Transplantation (1995) 59:772–7. 10.1097/00007890-199503150-00023 7886806

[90] Okumi M Fishbein JM Griesemer AD Gianello PR Hirakata A Nobori S Role of persistence of antigen and indirect recognition in the maintenance of tolerance to renal allografts. Transplantation (2008) 85:270–80. 10.1097/TP.0b013e31815e8eed 18212633 PMC2862307

[91] Hamano K Rawsthorne MA Bushell AR Morris PJ Wood KJ. Evidence that the continued presence of the organ graft and not peripheral donor microchimerism is essential for maintenance of tolerance to alloantigen in vivo in anti-CD4 treated recipients. Transplantation (1996) 62:856–60. 10.1097/00007890-199609270-00026 8824489

[92] Scalea JR Villani V Gillon BC Weiner J Gianello P Turcotte N Development of antidonor antibody directed toward non-major histocompatibility complex antigens in tolerant animals. Transplantation (2014) 98:514–9. 10.1097/TP.0000000000000249 24933456 PMC4149823

[93] Graca L. Transplantation tolerance: context matters. Eur J Immunol (2015) 45:1921–5. 10.1002/eji.201545762 26031651

[94] Chai JG Ratnasothy K Bucy RP Noelle RJ Lechler R Lombardi G. Allospecific CD4(+) T cells retain effector function and are actively regulated by Treg cells in the context of transplantation tolerance. Eur J Immunol (2015) 45:2017–27. 10.1002/eji.201545455 25944401

[95] Rosenblum MD Gratz IK Paw JS Lee K Marshak-Rothstein A Abbas AK. Response to self antigen imprints regulatory memory in tissues. Nature (2011) 480:538–42. 10.1038/nature10664 22121024 PMC3263357

[96] Warren KJ Iwami D Harris DG Bromberg JS Burrell BE. Laminins affect T cell trafficking and allograft fate. J Clin Invest (2014) 124:2204–18. 10.1172/JCI73683 24691446 PMC4001556

[97] Riquelme P Geissler EK Hutchinson JA. Alternative approaches to myeloid suppressor cell therapy in transplantation: comparing regulatory macrophages to tolerogenic DCs and MDSCs. Transplant Res (2012) 1:17. 10.1186/2047-1440-1-17 23369628 PMC3561050

[98] Hutchinson JA Riquelme P Geissler EK Fändrich F. Human regulatory macrophages. Methods Mol Biol (2011) 677:181–92. 10.1007/978-1-60761-869-0_13 20941611

[99] Riquelme P Tomiuk S Kammler A Fändrich F Schlitt HJ Geissler EK IFN-γ-induced iNOS expression in mouse regulatory macrophages prolongs allograft survival in fully immunocompetent recipients. Mol Ther (2013) 21:409–22. 10.1038/mt.2012.168 22929659 PMC3594012

[100] Hutchinson JA Riquelme P Sawitzki B Tomiuk S Miqueu P Zuhayra M Cutting edge: immunological consequences and trafficking of human regulatory macrophages administered to renal transplant recipients. J Immunol (2011) 187:2072–8. 10.4049/jimmunol.1100762 21804023

[101] Hutchinson JA Riquelme P Brem-Exner BG Schulze M Matthäi M Renders L Transplant acceptance-inducing cells as an immune-conditioning therapy in renal transplantation. Transpl Int (2008) 21:728–41. 10.1111/j.1432-2277.2008.00680.x 18573142

[102] Hutchinson JA Brem-Exner BG Riquelme P Roelen D Schulze M Ivens K A cell-based approach to the minimization of immunosuppression in renal transplantation. Transpl Int (2008) 21:742–54. 10.1111/j.1432-2277.2008.00692.x 18573141

[103] Hutchinson JA Roelen D Riquelme P Brem-Exner BG Witzke O Philipp T Preoperative treatment of a presensitized kidney transplant recipient with donor-derived transplant acceptance-inducing cells. Transpl Int (2008) 21:808–13. 10.1111/j.1432-2277.2008.00712.x 18573140

[104] Geissler EK. The ONE Study compares cell therapy products in organ transplantation: introduction to a review series on suppressive monocyte-derived cells. Transplant Res (2012) 1:11. 10.1186/2047-1440-1-10 23369457 PMC3561076

[105] Ezzelarab MB Zahorchak AF Lu L Morelli AE Chalasani G Demetris AJ Regulatory dendritic cell infusion prolongs kidney allograft survival in nonhuman primates. Am J Transplant (2013) 13:1989–2005. 10.1111/ajt.12310 23758811 PMC4070451

[106] Dhodapkar MV Steinman RM Krasovsky J Munz C Bhardwaj N. Antigen-specific inhibition of effector T cell function in humans after injection of immature dendritic cells. J Exp Med (2001) 193:233–8. 10.1084/jem.193.2.233 11208863 PMC2193335

[107] Dhodapkar MV Steinman RM. Antigen-bearing immature dendritic cells induce peptide-specific CD8(+) regulatory T cells in vivo in humans. Blood (2002) 100:174–7. 10.1182/blood.V100.1.174 12070024

[108] Giannoukakis N Phillips B Finegold D Harnaha J Trucco M. Phase I (safety) study of autologous tolerogenic dendritic cells in type 1 diabetic patients. Diabetes Care (2011) 34:2026–32. 10.2337/dc11-0472 21680720 PMC3161299

[109] Gabrilovich DI Nagaraj S. Myeloid-derived suppressor cells as regulators of the immune system. Nat Rev Immunol (2009) 9:162–74. 10.1038/nri2506 19197294 PMC2828349

[110] Bronte V Zanovello P. Regulation of immune responses by l-arginine metabolism. Nat Rev Immunol (2005) 5:641–54. 10.1038/nri1668 16056256

[111] Rodríguez PC Ochoa AC. Arginine regulation by myeloid derived suppressor cells and tolerance in cancer: mechanisms and therapeutic perspectives. Immunol Rev (2008) 222:180–91. 10.1111/j.1600-065X.2008.00608.x 18364002 PMC3546504

[112] Rodriguez PC Zea AH Culotta KS Zabaleta J Ochoa JB Ochoa AC. Regulation of T cell receptor CD3zeta chain expression by l-arginine. J Biol Chem (2002) 277:21123–9. 10.1074/jbc.M110675200 11950832

[113] Rodriguez PC Quiceno DG Ochoa AC. l-arginine availability regulates T-lymphocyte cell-cycle progression. Blood (2007) 109:1568–73. 10.1182/blood-2006-06-031856 17023580 PMC1794048

[114] Harari O Liao JK. Inhibition of MHC II gene transcription by nitric oxide and antioxidants. Curr Pharm Des (2004) 10:893–8. 10.2174/1381612043452893 15032692 PMC2633593

[115] Rivoltini L Carrabba M Huber V Castelli C Novellino L Dalerba P Immunity to cancer: attack and escape in T lymphocyte-tumor cell interaction. Immunol Rev (2002) 188:97–113. 10.1034/j.1600-065X.2002.18809.x 12445284

[116] Huang B Pan PY Li Q Sato AI Levy DE Bromberg J Gr-1+CD115+ immature myeloid suppressor cells mediate the development of tumor-induced T regulatory cells and T-cell anergy in tumor-bearing host. Cancer Res (2006) 66:1123–31. 10.1158/0008-5472.CAN-05-1299 16424049

[117] Hiramoto K Satoh H Suzuki T Moriguchi T Pi J Shimosegawa T Myeloid lineage-specific deletion of antioxidant system enhances tumor metastasis. Cancer Prev Res (Phila) (2014) 7:835–44. 10.1158/1940-6207.CAPR-14-0094 24866179

[118] Corzo CA Cotter MJ Cheng P Cheng F Kusmartsev S Sotomayor E Mechanism regulating reactive oxygen species in tumor-induced myeloid-derived suppressor cells. J Immunol (2009) 182:5693–701. 10.4049/jimmunol.0900092 19380816 PMC2833019

[119] Vickers SM MacMillan-Crow LA Green M Ellis C Thompson JA. Association of increased immunostaining for inducible nitric oxide synthase and nitrotyrosine with fibroblast growth factor transformation in pancreatic cancer. Arch Surg (1999) 134:245–51. 10.1001/archsurg.134.3.245 10088562

[120] Cobbs CS Whisenhunt TR Wesemann DR Harkins LE Van Meir EG Samanta M. Inactivation of wild-type p53 protein function by reactive oxygen and nitrogen species in malignant glioma cells. Cancer Res (2003) 63:8670–3. 14695179

[121] Nakamura Y Yasuoka H Tsujimoto M Yoshidome K Nakahara M Nakao K Nitric oxide in breast cancer: induction of vascular endothelial growth factor-C and correlation with metastasis and poor prognosis. Clin Cancer Res (2006) 12:1201–7. 10.1158/1078-0432.CCR-05-1269 16489074

[122] Yang R Cai Z Zhang Y Yutzy WH Roby KF Roden RB. CD80 in immune suppression by mouse ovarian carcinoma-associated Gr-1+CD11b+ myeloid cells. Cancer Res (2006) 66:6807–15. 10.1158/0008-5472.CAN-05-3755 16818658

[123] Serafini P Mgebroff S Noonan K Borrello I. Myeloid-derived suppressor cells promote cross-tolerance in B-cell lymphoma by expanding regulatory T cells. Cancer Res (2008) 68:5439–49. 10.1158/0008-5472.CAN-07-6621 18593947 PMC2887390

[124] Ambrosino E Spadaro M Iezzi M Curcio C Forni G Musiani P Immunosurveillance of Erbb2 carcinogenesis in transgenic mice is concealed by a dominant regulatory T-cell self-tolerance. Cancer Res (2006) 66:7734–40. 10.1158/0008-5472.CAN-06-1432 16885376

[125] Nagaraj S Youn JI Gabrilovich DI. Reciprocal relationship between myeloid-derived suppressor cells and T cells. J Immunol (2013) 191:17–23. 10.4049/jimmunol.1300654 23794702 PMC3694485

[126] Dugast AS Haudebourg T Coulon F Heslan M Haspot F Poirier N Myeloid-derived suppressor cells accumulate in kidney allograft tolerance and specifically suppress effector T cell expansion. J Immunol (2008) 180:7898–906. 10.4049/jimmunol.180.12.7898 18523253

[127] Dilek N Poirier N Usal C Martinet B Blancho G Vanhove B. Control of transplant tolerance and intragraft regulatory T cell localization by myeloid-derived suppressor cells and CCL5. J Immunol (2012) 188:4209–16. 10.4049/jimmunol.1101512 22450806

[128] Chou HS Hsieh CC Charles R Wang L Wagner T Fung JJ Myeloid-derived suppressor cells protect islet transplants by B7-H1 mediated enhancement of T regulatory cells. Transplantation (2012) 93:272–82. 10.1097/TP.0b013e31823ffd39 22179405 PMC3267010

[129] De Wilde V Van Rompaey N Hill M Lebrun JF Lemaître P Lhommé F Endotoxin-induced myeloid-derived suppressor cells inhibit alloimmune responses via heme oxygenase-1. Am J Transplant (2009) 9:2034–47. 10.1111/j.1600-6143.2009.02757.x 19681826

[130] Zahorchak AF Ezzelarab MB Lu L Turnquist HR Thomson AW In vivo mobilization and functional characterization of nonhuman primate monocytic myeloid-derived suppressor cells. Am J Transplant (2016) 16(2):661–71. 26372923 10.1111/ajt.13454 PMC6521707

[131] Dilek N Vuillefroy de Silly R Blancho G Vanhove B. Myeloid-derived suppressor cells: mechanisms of action and recent advances in their role in transplant tolerance. Front Immunol (2012) 3:208. 10.3389/fimmu.2012.00208 22822406 PMC3398399

[132] Kim JI Rothstein DM Markmann JF. Role of B cells in tolerance induction. Curr Opin Organ Transplant (2015) 20:369–75. 10.1097/MOT.0000000000000204 26107970 PMC4517180

[133] Sicard A Koenig A Morelon E Defrance T Thaunat O. Cell therapy to induce allograft tolerance: time to switch to plan B? Front Immunol (2015) 6:149. 10.3389/fimmu.2015.00149 25904913 PMC4387960

[134] Wolf SD Dittel BN Hardardottir F Janeway CA. Experimental autoimmune encephalomyelitis induction in genetically B cell-deficient mice. J Exp Med (1996) 184:2271–8. 10.1084/jem.184.6.2271 8976182 PMC2196394

[135] Mauri C Gray D Mushtaq N Londei M. Prevention of arthritis by interleukin 10-producing B cells. J Exp Med (2003) 197:489–501. 10.1084/jem.20021293 12591906 PMC2193864

[136] Mizoguchi A Mizoguchi E Smith RN Preffer FI Bhan AK. Suppressive role of B cells in chronic colitis of T cell receptor alpha mutant mice. J Exp Med (1997) 186:1749–56. 10.1084/jem.186.10.1749 9362534 PMC2199135

[137] Amu S Saunders SP Kronenberg M Mangan NE Atzberger A Fallon PG. Regulatory B cells prevent and reverse allergic airway inflammation via FoxP3-positive T regulatory cells in a murine model. J Allergy Clin Immunol (2010) 125:1114.e–24.e. 10.1016/j.jaci.2010.01.018 20304473

[138] Braza F Chesne J Castagnet S Magnan A Brouard S. Regulatory functions of B cells in allergic diseases. Allergy (2014) 69:1454–63. 10.1111/all.12490 25060230

[139] Deng S Moore DJ Huang X Lian MM Mohiuddin M Velededeoglu E Cutting edge: transplant tolerance induced by anti-CD45RB requires B lymphocytes. J Immunol (2007) 178:6028–32. 10.4049/jimmunol.178.10.6028 17475825

[140] Lee KM Kim JI Stott R Soohoo J O’Connor MR Yeh H Anti-CD45RB/anti-TIM-1-induced tolerance requires regulatory B cells. Am J Transplant (2012) 12:2072–8. 10.1111/j.1600-6143.2012.04055.x 22494812 PMC3396747

[141] Lee KM Stott RT Zhao G SooHoo J Xiong W Lian MM TGF-β-producing regulatory B cells induce regulatory T cells and promote transplantation tolerance. Eur J Immunol (2014) 44:1728–36. 10.1002/eji.201344062 24700192 PMC4048633

[142] Ding Q Yeung M Camirand G Zeng Q Akiba H Yagita H Regulatory B cells are identified by expression of TIM-1 and can be induced through TIM-1 ligation to promote tolerance in mice. J Clin Invest (2011) 121:3645–56. 10.1172/JCI46274 21821911 PMC3163958

[143] Mann MK Maresz K Shriver LP Tan Y Dittel BN. B cell regulation of CD4+CD25+ T regulatory cells and IL-10 via B7 is essential for recovery from experimental autoimmune encephalomyelitis. J Immunol (2007) 178:3447–56. 10.4049/jimmunol.178.6.3447 17339439

[144] Kessel A Haj T Peri R Snir A Melamed D Sabo E Human CD19(+)CD25(high) B regulatory cells suppress proliferation of CD4(+) T cells and enhance Foxp3 and CTLA-4 expression in T-regulatory cells. Autoimmun Rev (2012) 11:670–7. 10.1016/j.autrev.2011.11.018 22155204

[145] Tretter T Venigalla RK Eckstein V Saffrich R Sertel S Ho AD Induction of CD4+ T-cell anergy and apoptosis by activated human B cells. Blood (2008) 112:4555–64. 10.1182/blood-2008-02-140087 18802006

[146] Duddy M Niino M Adatia F Hebert S Freedman M Atkins H Distinct effector cytokine profiles of memory and naive human B cell subsets and implication in multiple sclerosis. J Immunol (2007) 178:6092–9. 10.4049/jimmunol.178.10.6092 17475834

[147] Blair PA Noreña LY Flores-Borja F Rawlings DJ Isenberg DA Ehrenstein MR CD19(+)CD24(hi)CD38(hi) B cells exhibit regulatory capacity in healthy individuals but are functionally impaired in systemic Lupus Erythematosus patients. Immunity (2010) 32:129–40. 10.1016/j.immuni.2009.11.009 20079667

[148] Flores-Borja F Bosma A Ng D Reddy V Ehrenstein MR Isenberg DA CD19+CD24hiCD38hi B cells maintain regulatory T cells while limiting TH1 and TH17 differentiation. Sci Transl Med (2013) 5:173ra23. 10.1126/scitranslmed.3005407 23427243

[149] Dong HP Elstrand MB Holth A Silins I Berner A Trope CG NK- and B-cell infiltration correlates with worse outcome in metastatic ovarian carcinoma. Am J Clin Pathol (2006) 125:451–8. 10.1309/15B66DQMFYYM78CJ 16613351

[150] Silva HM Takenaka MC Moraes-Vieira PM Monteiro SM Hernandez MO Chaara W Preserving the B-cell compartment favors operational tolerance in human renal transplantation. Mol Med (2012) 18:733–43. 10.2119/molmed.2011.00281 22252714 PMC3409285

[151] Brouard S Mansfield E Braud C Li L Giral M Hsieh SC Identification of a peripheral blood transcriptional biomarker panel associated with operational renal allograft tolerance. Proc Natl Acad Sci USA (2007) 104:15448–53. 10.1073/pnas.0705834104 17873064 PMC2000539

[152] Newell KA Asare A Kirk AD Gisler TD Bourcier K Suthanthiran M Identification of a B cell signature associated with renal transplant tolerance in humans. J Clin Invest (2010) 120:1836–47. 10.1172/JCI39933 20501946 PMC2877933

[153] Sagoo P Perucha E Sawitzki B Tomiuk S Stephens DA Miqueu P Development of a cross-platform biomarker signature to detect renal transplant tolerance in humans. J Clin Invest (2010) 120:1848–61. 10.1172/JCI39922 20501943 PMC2877932

[154] Thaunat O. Pathophysiologic significance of B-cell clusters in chronically rejected grafts. Transplantation (2011) 92:121–6. 10.1097/TP.0b013e31821f74fe 21555973

[155] Scott DW Zhang AH Su Y. B-cell based gene therapy for autoimmune diseases. Infect Disord Drug Targets (2012) 12:241–7. 10.2174/187152612800564383 22394178

[156] Liang W Karabekian Z Mattapallil M Xu Q Viley AM Caspi R B-cell delivered gene transfer of human S-Ag-Ig fusion protein protects from experimental autoimmune uveitis. Clin Immunol (2006) 118:35–41. 10.1016/j.clim.2005.08.007 16168712

[157] Melo ME Qian J El-Amine M Agarwal RK Soukhareva N Kang Y Gene transfer of Ig-fusion proteins into B cells prevents and treats autoimmune diseases. J Immunol (2002) 168:4788–95. 10.4049/jimmunol.168.9.4788 11971030

[158] Xu B Scott DW. A novel retroviral gene therapy approach to inhibit specific antibody production and suppress experimental autoimmune encephalomyelitis induced by MOG and MBP. Clin Immunol (2004) 111:47–52. 10.1016/j.clim.2003.12.013 15093551

[159] Soukhareva N Jiang Y Scott DW. Treatment of diabetes in NOD mice by gene transfer of Ig-fusion proteins into B cells: role of T regulatory cells. Cell Immunol (2006) 240:41–6. 10.1016/j.cellimm.2006.06.004 16860296

[160] Satpute SR Soukhareva N Scott DW Moudgil KD. Mycobacterial Hsp65-IgG-expressing tolerogenic B cells confer protection against adjuvant-induced arthritis in Lewis rats. Arthritis Rheum (2007) 56:1490–6. 10.1002/art.22566 17469108

[161] Skupsky J Zhang AH Su Y Scott DW. B-cell-delivered gene therapy induces functional T regulatory cells and leads to a loss of antigen-specific effector cells. Mol Ther (2010) 18:1527–35. 10.1038/mt.2010.95 20485267 PMC2927073

[162] Calderón-Gómez E Lampropoulou V Shen P Neves P Roch T Stervbo U Reprogrammed quiescent B cells provide an effective cellular therapy against chronic experimental autoimmune encephalomyelitis. Eur J Immunol (2011) 41:1696–708. 10.1002/eji.201041041 21469107 PMC3431508

[163] Poon IK Lucas CD Rossi AG Ravichandran KS. Apoptotic cell clearance: basic biology and therapeutic potential. Nat Rev Immunol (2014) 14:166–80. 10.1038/nri3607 24481336 PMC4040260

[164] Saas P Kaminski S Perruche S. Prospects of apoptotic cell-based therapies for transplantation and inflammatory diseases. Immunotherapy (2013) 5:1055–73. 10.2217/imt.13.103 24088076

[165] Bonnefoy F Perruche S Couturier M Sedrati A Sun Y Tiberghien P Plasmacytoid dendritic cells play a major role in apoptotic leukocyte-induced immune modulation. J Immunol (2011) 186:5696–705. 10.4049/jimmunol.1001523 21460208

